# Multiple sites in the N-terminal half of simian immunodeficiency virus capsid protein contribute to evasion from rhesus monkey TRIM5α-mediated restriction

**DOI:** 10.1186/1742-4690-7-72

**Published:** 2010-09-08

**Authors:** Ken Kono, Haihan Song, Masaru Yokoyama, Hironori Sato, Tatsuo Shioda, Emi E Nakayama

**Affiliations:** 1 Department of Viral Infections, Research Institute for Microbial Diseases, Osaka University, 3-1 Yamada-oka, Suita, Osaka 565-0871, Japan; 2 Laboratory of Viral Genomics, Pathogen Genomics Center, National Institute of Infectious Diseases, Gakuen 4-7-1, MusashiMurayama-shi, Tokyo, 208-0011, Japan

## Abstract

**Background:**

We previously reported that cynomolgus monkey (CM) TRIM5α could restrict human immunodeficiency virus type 2 (HIV-2) strains carrying a proline at the 120^th ^position of the capsid protein (CA), but it failed to restrict those with a glutamine or an alanine. In contrast, rhesus monkey (Rh) TRIM5α could restrict all HIV-2 strains tested but not simian immunodeficiency virus isolated from macaque (SIVmac), despite its genetic similarity to HIV-2.

**Results:**

We attempted to identify the viral determinant of SIVmac evasion from Rh TRIM5α-mediated restriction using chimeric viruses formed between SIVmac239 and HIV-2 GH123 strains. Consistent with a previous study, chimeric viruses carrying the loop between α-helices 4 and 5 (L4/5) (from the 82^nd ^to 99^th ^amino acid residues) of HIV-2 CA were efficiently restricted by Rh TRIM5α. However, the corresponding loop of SIVmac239 CA alone (from the 81^st ^to 97^th ^amino acid residues) was not sufficient to evade Rh TRIM5α restriction in the HIV-2 background. A single glutamine-to-proline substitution at the 118^th ^amino acid of SIVmac239 CA, corresponding to the 120^th ^amino acid of HIV-2 GH123, also increased susceptibility to Rh TRIM5α, indicating that glutamine at the 118^th ^of SIVmac239 CA is necessary to evade Rh TRIM5α. In addition, the N-terminal portion (from the 5^th ^to 12^th ^amino acid residues) and the 107^th ^and 109^th ^amino acid residues in α-helix 6 of SIVmac CA are necessary for complete evasion from Rh TRIM5α-mediated restriction. A three-dimensional model of hexameric GH123 CA showed that these multiple regions are located on the CA surface, suggesting their direct interaction with TRIM5α.

**Conclusion:**

We found that multiple regions of the SIVmac CA are necessary for complete evasion from Rh TRIM5α restriction.

## Background

The host range of human immunodeficiency virus type 1 (HIV-1) is very narrow, being limited to humans and chimpanzees [1]. HIV-1 fails to replicate in activated CD4-positive T lymphocytes obtained from Old World monkeys (OWM) such as rhesus (Rh) [2,3] and cynomolgus (CM) monkeys [4,5]. Simian immunodeficiency virus (SIV) isolated from sooty mangabey (SIVsm) and SIV isolated from African green monkey (SIVagm) replicate in their natural hosts [6]. SIV isolated from a macaque monkey (SIVmac) evolved from SIVsm in captive macaques, and replicates efficiently in Rh [2,3] and CM [4,5] monkeys. Human immunodeficiency virus type 2 (HIV-2) is assumed to have originated from SIVsm as the result of zoonotic events involving monkeys and humans [7]. Previous studies have shown that HIV-2 strains vary widely in their ability to grow in cells of OWM such as baboon, and Rh and CM monkeys [8-12].

In 2004, the screening of a Rh cDNA library identified TRIM5α as a factor that confers resistance to HIV-1 infection [13]. Both Rh and CM TRIM5α proteins restrict HIV-1 infection but fail to restrict SIVmac [13,14]. In contrast, human TRIM5α is almost powerless to restrict the aforementioned viruses, but potently restricts N-tropic murine leukemia viruses (N-MLV) and equine infectious anemia virus [15-17].

TRIM5α is a member of the tripartite motif (TRIM) family of proteins, and consists of RING, B-box 2, coiled-coil, and SPRY (B30.2) domains [18]. Proteins with RING domains possess E3 ubiquitin ligase activity [19]; therefore, TRIM5α was thought to restrict HIV-1 by proteasome-dependent pathways. However, proteasome inhibitors do not affect TRIM5α-mediated HIV-1 restriction, even though HIV-1 late reverse transcribed products are generated normally [20-22]. TRIM5α is thus supposed to use both proteasome-dependent and -independent pathways to restrict HIV-1.

The intact B-box 2 domain is also required for TRIM5α-mediated antiviral activity, since TRIM5α restrictive activity is diminished by several amino acid substitutions in the B-box 2 domain [23,24]. TRIM5α has been shown to form a dimer [25,26], while the B-box 2 domain mediates higher-order self-association of Rh TRIM5α oligomers [27,28]. The coiled-coil domain of TRIM5α is important for the formation of homo-oligomers [29], and the homo-oligomerization of TRIM5α is essential for antiviral activity [30,31]. The SPRY domain is specific for an α-isoform among at least three splicing variants transcribed from the *TRIM5 *gene. Soon after the identification of TRIM5α as a restriction factor of Rh, several studies found that differences in the amino acid sequences of the TRIM5α SPRY domain of different monkey species affect the species-specific restriction of retrovirus infection [14,32-39]. Studies on human and Rh recombinant TRIM5αs have shown that the determinant of species-specific restriction against HIV-1 infection resides in variable region 1 (V1) of the SPRY domain [32,33]. In the case of HIV-2 infection, we previously found that three amino acid residues of TFP at the 339^th ^to 341^st ^positions of Rh TRIM5α V1 are indispensable for restricting particular HIV-2 strains that are still resistant to CM TRIM5α [34].

The SPRY domain is thus thought to recognize viral cores. Biochemical studies have shown that TRIM5α associates with CA in detergent-stripped N-MLV virions [40] or with an artificially constituted HIV-1 core structure composed of the capsid-nucleocapsid (CA-NC) fusion protein in a SPRY domain-dependent manner [41]. Ylinen *et al*. mapped one of the determinants of Rh TRIM5α sensitivity to a loop between α-helices 4 and 5 (L4/5) of HIV-2 [42]. In the present study, we found that the 120^th ^amino acid of HIV-2 CA, which is the determinant of CM TRIM5α sensitivity, also contributes to Rh TRIM5α susceptibility. Furthermore, studies on chimeric viruses between Rh TRIM5α-sensitive HIV-2 and -resistant SIVmac revealed that multiple regions in the N-terminal half of SIVmac CA including L4/5 contribute to the escape of SIVmac from Rh TRIM5α.

## Methods

### DNA constructs

The HIV-2 derivatives were constructed on a background of infectious molecular clone GH123 [43]. Construction of GH123/Q, the mutant GH123 possessing Q at the 120^th ^position of CA protein, and SIVmac239/P, the mutant SIVmac239 possessing P at the 118^th ^position of CA, were described previously [44]. The CA L4/5 of GH123 or GH123/Q was replaced with the corresponding segments of SIVmac239 CA using site-directed mutagenesis with the PCR-mediated overlap primer extension method [45], and the resultant constructs were designated GH123/CypS or GH123/CypS 120Q, respectively. The GH123 derivative with L4/5 of SIVmac239, Q at the 120^th^, and A at the 179^th ^position of CA (GH123/CypS 120Q 179A) was generated by site-directed mutagenesis on a background of GH123/CypS 120Q.

Chimeric GH123 containing the whole region of SIVmac239 CA (GH/SCA) was generated by site-directed mutagenesis. Restriction enzyme sites *Ngo*M IV and *Xho *I, located in the LTR and p6 cording region, respectively, were used for DNA recombination. To obtain the *Ngo*M IV-*Xho *I fragment containing the CA region, we performed four successive PCR reactions using GH123 and SIVmac239 as templates. The primers used in these reactions were GH114F (5'-TTGGCCGGCACTGG-3'), SCA1For (5'-CCAGTACAACAAATAGG-3'), SCA1 Rev (5'-CCTATTTGTTGTACTGG-3'), SCA2 For (5'-GCTAGATTAATGGCCGAAGCCCTG-3'), SCA2 Rev (5'-CAGGGCTTCGGCCATTAATCTAGC-3'), and 2082R (5'-GACAGAGGACTTGCTGCAC-3').

The first PCR reaction used GH123 as a template and GH114F and GHSCA1 Rev as primers, the second used SIVmac239 as a template and GHSCA1 For and GHSCA2 Rev as primers, and the third used GH123 as a template and GHSCA2 For and 2082R as primers. The resultant 1^st^, 2^nd^, and 3^rd ^fragments were used as templates in the fourth reaction with GH114F and 2082R as primers. The resultant *Ngo*M IV-*Xho *I fragment was transferred to GH123. GH/SCA derivatives GH/SCA N-G, GH/SCA VD, GH/SCA CypG, and GH/SCA TE were constructed by site-directed mutagenesis on a GH/SCA background.

To construct GH/NSCG, a GH123 derivative containing the N-terminal half (from 1^st ^to 120^th^) of SIVmac239CA, we performed three successive PCR reactions. The first used GH/SCA as a template and GH114F and NSCA Rev (5'-GGGATTTTGTTGTCTGTACATCC-3') as primers, the second used GH123 as a template and NSCA For (5'-GGATGTACAGACAACAAAATCCC-3') and 2082R as primers. The resultant 1^st ^and 2^nd ^fragments were used as templates in the third reaction with GH114F and 2082R as primers. The resultant *Ngo*M IV-*Xho *I fragment was transferred to GH123. The GH/NSCG derivative GH/GSG was constructed by site-directed mutagenesis on a GH/NSCG background.

### Cells

The 293T (human kidney) and FRhK4 (Rh kidney; American Type Culture Collection, Manassas, VA) were cultured in Dulbecco's modified Eagle medium supplemented with 10% heat-inactivated fetal bovine serum (FBS). MT4, a human CD4 positive T cell line immortalized by human T cell leukemia virus type 1 [46], was maintained in RPMI 1640 medium containing 10% FBS.

### Viral propagation

Virus stocks were prepared by transfection of 293T cells with HIV-2 GH123 derivatives using the calcium phosphate co-precipitation method. Viral titers were measured with the p27 RETROtek antigen ELISA kit (ZeptoMetrix, Buffalo, NY).

Recombinant Sendai virus (SeV) carrying Rh, CM, or CM SPRY(-) TRIM5α was described previously [14,34]. Green fluorescence protein (GFP) expressing HIV-1 carrying SIVmac239 L4/5 (HIV-1-L4/5-GFP) was prepared as described previously [47].

### Viral infection

MT4 cells (2 × 10^5^) were infected with SeV expressing each of the TRIM5αs, at a multiplicity of infection (MOI) of 10 plaque-forming units (pfu) per cell and incubated at 37°C for 9 h. Cells were then superinfected with 20 ng of p25 of HIV-2 GH123 or derivatives, or 20 ng of p27 of SIVmac239 or derivatives. Culture supernatants were collected periodically, and the levels of p25 or p27 were measured with the RETROtek antigen ELISA kit.

### Particle purification and Western blot analysis

Culture supernatant of 293T cells transfected with plasmids encoding HIV-1 NL43 and HIV-2 GH123 derivatives was clarified using low-speed centrifugation. The resultant supernatants were layered onto a cushion of 20% sucrose (made in PBS) and centrifuged at 35,000 rpm for 2 h in a Beckman SW41 rotor. After centrifugation, the virion pellets were resuspended in PBS and applied to sodium dodecyl sulfate-polyacrylamide gel electrophoresis (SDS-PAGE). Virion-associated proteins were transferred to a PVDF membrane. CAs and cyclophilin A (CypA) were visualized with the serum from SIV-infected monkeys or the anti-CypA antibody (Affinity BioReagents, Golden, CO), respectively.

### Saturation assay

HIV-2 or SIVmac derivative particles were prepared by co-transfection of the relevant plasmids with one encoding vesicular stomatitis virus glycoprotein (VSV-G) into 293T cells, and culture supernatants were collected two days after transfection. One day before infection, FRhK-4 cells were plated at a density of 2 × 10^4 ^cells per well in a 24-well plate. Prior to GFP virus infection, the cells were pretreated for 2 h with 800 ng of p25 of each of HIV-2 or SIVmac derivatives pseudotyped with VSV-G. Immediately after pretreatment, cells were washed and infected with 10 ng of p24 of the HIV-1-L4/5-GFP virus. Then, 2 h after infection, the inoculated GFP viruses were washed and the cells cultivated in fresh media. Two days after infection, GFP-positive cells were counted with a flow cytometer.

### Molecular modeling of hexameric HIV-2 CA

The crystal structures of the HIV-2 CA N-terminal domain at a resolution of 1.25Å [PDB: 2WLV] [48], HIV-1 CA C-terminal domain at a resolution of 1.70Å (PDB code: 1A8O) [49], and hexameric HIV-1 CA at a resolution of 1.90Å [PDB:3H47] [50] were taken from the RCSB Protein Data Bank [51]. Three-dimensional (3-D) models of monomeric HIV-2 CA were constructed by the homology modeling technique using 'MOE-Align' and 'MOE-Homology' in the Molecular Operating Environment (MOE) version 2008.1002 (Chemical Computing Group Inc., Quebec, Canada) as described [44,52]. We obtained 25 intermediate models per one homology modeling in MOE, and selected those 3-D models which were intermediate with best scores according to the generalized Born/volume integral methodology [53]. The final 3-D models were thermodynamically optimized by energy minimization using an AMBER99 force field [54] combined with the generalized Born model of aqueous solvation implemented in MOE [55]. Physically unacceptable local structures of the optimized 3-D models were further refined on the basis of evaluation by the Ramachandran plot using MOE. The structures of hexameric HIV-2 CA were generated from the monomeric structures by MOE on the basis of the assembly information of hexameric HIV-1 CA crystal structures [50].

## Results

### The L4/5 loop of SIVmac239 CA and Q and A at the 120^th ^and 179^th ^positions of CA are not sufficient for HIV-2 to evade Rh TRIM5α-mediated restriction

**Figure 1:**
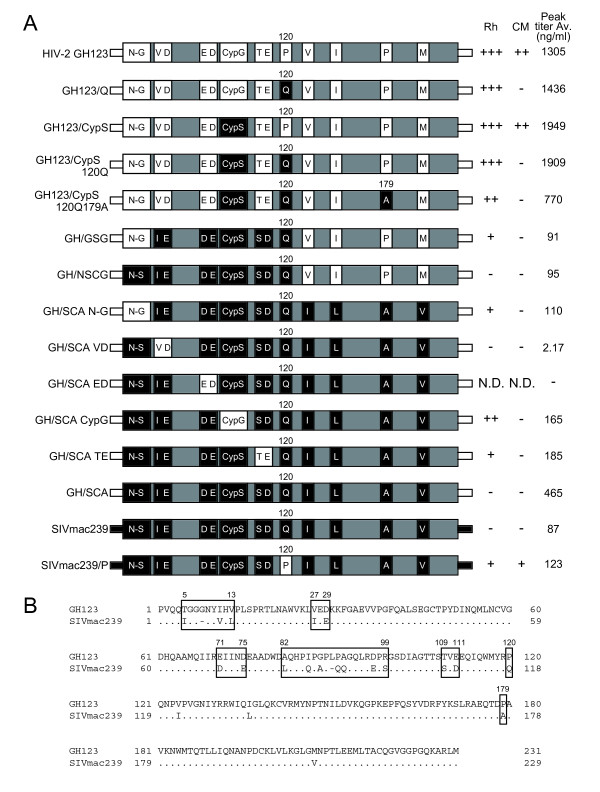
**Schematic representation of chimeric viral CAs**. (A) White and black bars denote HIV-2 GH123 and SIVmac239 sequences, respectively. +++, ++, +, and - denote more than 1000-fold, 100- to 1000-fold, 5- to 100-fold, and less than 5-fold suppression of viral growth, respectively, compared with viral growth in the presence of negative control CM SPRY(-) TRIM5α on day 6. Peak titer Av. denotes average titers in the presence of CM SPRY(-) TRIM5α on day 6 of two independent experiments. (B) Alignments of amino acid sequences of GH123 and SIVmac239 CAs. Dots denote amino acid residues identical to one of the GH123 CA and dashes denote lack of an amino acid residue present in GH123 CA. Boxes show the regions replaced between GH123 and SIVmac239.

**Figure 2:**
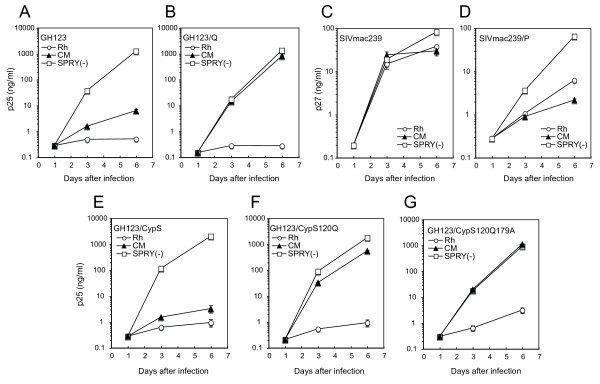
**MT4 cells were infected with recombinant SeV expressing Rh (white circles), CM (black triangles), or CM SPRY(-) (white squares) TRIM5α**. Nine hours after infection, cells were superinfected with GH123, SIVmac239 or their derivative viruses. Culture supernatants were separately assayed for levels of p25 from GH123 or p27 from SIVmac239. Error bars show actual fluctuations between levels of p25 or p27 in duplicate samples. A representative of two independent experiments is shown.

Previously, we evaluated the antiviral effect of CM and Rh TRIM5α and found that CM TRIM5α could restrict HIV-2 GH123 carrying P at the 120^th ^position of CA, but failed to restrict the HIV-2 GH123 mutant in which P was replaced with Q (GH123/Q) [44] (Figure 1A). In contrast, Rh TRIM5α could restrict both viruses [34] (Figure 2A and 2B). Although CA of HIV-2 GH123 and SIVmac239 share more than 87% amino acid identity (Figure 1B), CM and Rh TRIM5αs failed to restrict SIVmac239 (Figure 2C).

Since wild type SIVmac239 possesses Q at the 118^th ^position of CA (analogous to the 120^th ^position of GH123 CA), we constructed mutant SIVmac239 carrying P at the 118^th ^position (SIVmac239/P), and found that CM and Rh TRIM5αs could restrict the mutant virus [44] (Figure 2D). These results indicate that Q at the 118^th ^position of CA is required to evade restriction by CM and Rh TRIM5αs, although Rh TRIM5α could restrict GH123/Q. In the case of Rh TRIM5α, it has been reported that Rh TRIM5α sensitivity determinants lie in the loop between α-helices 4 and 5 of CA protein, equivalent to the cyclophilin A (CypA) binding loop of HIV-1 [42]. This conclusion was made after Rh TRIM5α restricted SIVmac-based SIV H2L in which the L4/5 was replaced with that of HIV-2. However, when we constructed a GH123 derivative in which L4/5 was replaced with that of SIVmac239 (GH123/CypS), the reciprocal virus of SIV H2L, we found that Rh TRIM5α still restricted this virus very well (Figure 2E), indicating that SIVmac239 L4/5 alone is not sufficient for HIV-2 to evade Rh TRIM5α restriction.

We then constructed a GH123 derivative with L4/5 of SIVmac239 (CypS) and Q at the 120^th ^position of CA (GH123/CypS 120Q). Contrary to our expectations, Rh TRIM5α still fully restricted this virus (Figure 2F). Since we previously found that the amino acid change at the 179^th ^position of HIV-2 CA correlated with plasma viral load in infected individuals [56], we next replaced P at the 179^th ^position of GH123/CypS 120Q CA with alanine (A) of SIVmac239 CA analogous to the 179^th ^position of GH123 CA to generate GH123/CypS 120Q179A. However, Rh TRIM5α also completely restricted this virus (Figure 2G). The peak titers of GH123/CypS 120Q and GH123/CypS 120Q179A in cells expressing Rh TRIM5α were approximately 1000 times (+++ in Figure 1) and 300 times (++ in Figure 1), respectively, lower than those in cells expressing CM TRIM5α lacking the SPRY domain, CM SPRY (-) TRIM5α, a negative control for functional TRIM5α (Figure 2F and 2G). Although this result suggests that the 179^th ^amino acid slightly contributes to evade Rh TRIM5α, it is clear that L4/5 of SIVmac239 CA and Q at the 120^th ^and A at the 179^th ^positions of CA were insufficient to evade Rh TRIM5α-mediated restriction.

In the case of CM TRIM5α, viruses carrying P at the 120^th ^position (GH123, GH123/CypS, and SIVmac239/P) were restricted by CM TRIM5α, whereas all other viruses bearing Q (GH123/Q, GH123/CypS 120Q, GH123/CypS 120Q179A, and SIVmac239) were not (Figures 1 and 2). These results are in good agreement with our previous conclusion that glutamine at the 120^th ^position of HIV-2 CA alone is sufficient to evade CM TRIM5α restriction [34,44].

### The N-terminal half of SIVmac239 CA is sufficient to evade Rh TRIM5α

**Figure 3:**
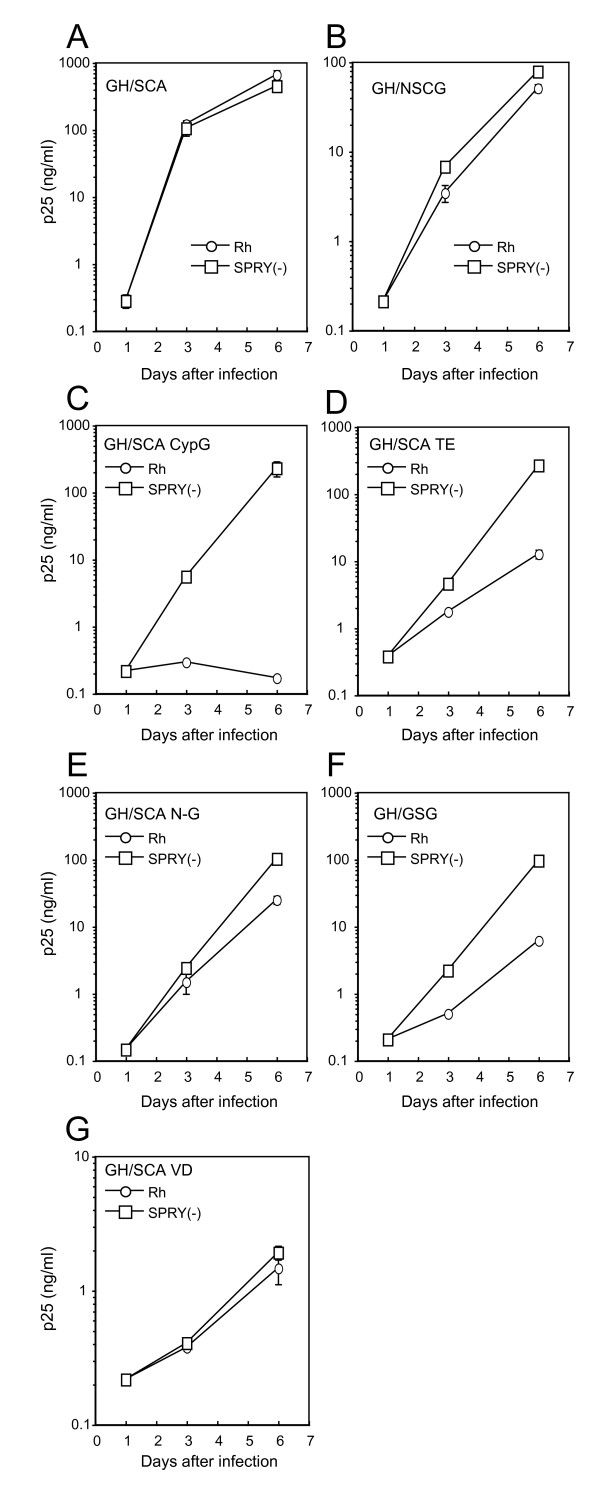
**MT4 cells were infected with recombinant SeV expressing Rh (white circles) or CM SPRY(-) (white squares) TRIM5α**. Nine hours after infection, cells were superinfected with GH/SCA (A), GH/NSCG (B) or GH/SCA derivatives (C-G). Culture supernatants were separately assayed for levels of p25. Error bars show actual fluctuations between levels of p25 in duplicate samples. A representative of two independent experiments is shown.

To confirm that CA contains all determinants for restriction by Rh TRIM5α, we constructed a chimeric GH123 containing the whole region of SIVmac239 CA (GH/SCA). This virus could grow in the presence and absence of Rh TRIM5α (Figures 1 and 3A), clearly excluding the possibility that some of the determinants lie outside the CA. We then generated a chimeric GH123 containing the N-terminal half (from the 1^st ^to 120^th^) of SIVmac239 CA (GH/NSCG) to further narrow down the determinant for restriction by Rh TRIM5α. Although GH/NSCG grew to lower titers than GH/SCA, even in the absence of Rh TRIM5α, this virus could also grow in the presence of Rh TRIM5α (Figures 1 and 3B). These results suggest that the N-terminal half of SIVmac239 CA is almost sufficient to evade Rh TRIM5α, even though the 179^th ^amino acid of the C-terminal half possessed a slight effect of restriction.

### Multiple sites in the N-terminal half of SIVmac239 CA contribute to evasion from restriction by Rh TRIM5α

In the N-terminal half of GH123 CA, 19 amino acid residues differ from those of SIVmac239. We grouped these differences into six regions as shown by boxes in Figure 1B, and evaluated their contribution to evasion from Rh TRIM5α by replacing each region of GH/SCA with the corresponding region of GH123. Rh TRIM5α completely restricted the GH/SCA derivative with the GH123 L4/5 (CypG) (GH/SCA CypG) (Figures 1 and 3C), consistent with a previous study [42]. Rh TRIM5α moderately restricted the GH/SCA derivative with threonine (T) and glutamic acid (E) of GH123 at the 109^th ^and 111^th ^positions, respectively (GH/SCA TE) (Figures 1 and 3D). These results suggest that not only L4/5 but also the 107^th ^and 109^th ^of amino acid residues of SIVmac239 CA (analogous to the 109^th ^and 111^th ^of GH123 CA) contribute to evasion from restriction by Rh TRIM5α.

Moreover, Rh TRIM5α slightly but significantly restricted the GH/SCA derivative with the GH123 N-terminal portion from the 5^th ^to 13^th ^amino acid residues (N-G) (GH/SCA N-G) (Figures 1 and 3E) (*p *< 0.05, t-test, n = 4), indicating that the SIVmac239 N-terminal portion from 5^th ^to 12^th ^(N-S) (analogous to N-G) is also important in evasion from Rh TRIM5α. Consistent with this result, Rh TRIM5α which failed to restrict GH/NSCG, could restrict the GH/NSCG derivative with N-G (GH/GSG) (Figures 1 and 3F). On the other hand, Rh TRIM5α failed to restrict the GH/SCA derivative with the valine (V) and aspartic acid (D) of GH123 at the 27^th ^and 29^th ^positions, respectively (GH/SCA VD) (Figures 1 and 3G). It should be noted, however, that the growth capability of GH/SCA VD in MT4 cells was extremely low even in the absence of TRIM5α (Figure 3G), and further studies are necessary to address the contribution of this region to viral sensitivity to Rh TRIM5α. Similarly, the GH/SCA derivative with glutamic acid (E) and D of GH123 at the 71^st ^and 75^th ^positions (GH/SCA ED) (Figure 1) did not grow in MT4 cells expressing CM SPRY (-) TRIM5α, thus, we were unable to evaluate the effect of these sites. Taken together, we conclude that multiple sites in the N-terminal half of SIVmac239 CA (N-S, CypS (L4/5), and the 107^th^, 109^th^, and 118^th ^amino acid residues) contribute to evasion from restriction by Rh TRIM5α.

We previously reported that a mutant CM TRIM5α possessing TFP instead of Q at the 339^th ^position (CM Q-TFP TRIM5α) potently restricted GH123/Q [34]. In the present study, CM Q-TFP TRIM5α showed nearly the same spectrum of virus restriction as Rh TRIM5α as it completely restricted GH/SCA CypG, moderately restricted GH/SCA TE and SIVmac239/P, and only slightly restricted GH/SCA N-G (data not shown). These results indicate that the virus restriction specificity of Rh TRIM5α is highly dependent on the three amino acid residues 339^th^-TFP-341^st^.

### CypA was not incorporated into GH123, SIVmac239 or their derivative virus particles

**Figure 4:**
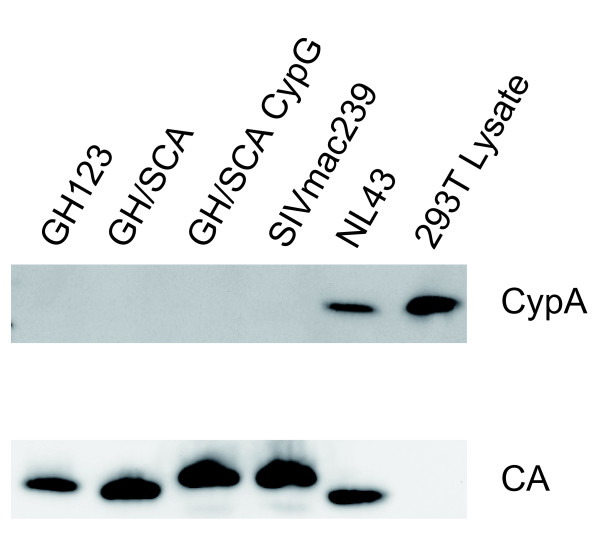
**Western blot analysis of CA and CypA in particles of GH123, SIVmac239 and their derivatives**. Viral particles from HIV-1 NL43, HIV-2 GH123, SIVmac239, and their derivatives were purified by ultracentrifugation through a 20% sucrose cushion. A total of 120 ng of p24 of HIV-1, p25 of HIV-2 GH123 derivatives or p27 of SIVmac239 derivatives was applied for gel electropholesis. Cyp A (upper panel) and CA (lower panel) were visualized by Western blotting (WB) using an anti-CypA antibody and serum from a SIV-infected monkey, respectively.

It has been reported that CypA was incorporated into group M HIV-1, but not HIV-2 or SIVmac particles [57]. To confirm that the replacement of CA between GH123 and SIVmac239 did not augment CypA incorporation, we performed Western blot analysis of viral particles from GH123, SIVmac239, and their derivatives. As shown in Figure 4 (upper panel), CypA proteins were clearly detected in the particles of HIV-1 NL43 but not in those of GH123, GH/SCA, GH/SCA CypG or SIVmac239, although the amount of their CA proteins was almost comparable (Figure 4, lower panel). This result indicates that the replacement between GH123 and SIVmac239 did not augment their CypA incorporation ability.

### Rh TRIM5α-resistant HIV-2 derivative virions showed impaired saturation activity to TRIM5α in Rh cells

It is known that TRIM5α-mediated restriction of retroviral infection is saturated when cells are exposed to high doses of restriction-sensitive viral particles [58-61]. To determine whether the amino acid substitutions we generated would affect the viral ability to saturate TRIM5α restriction, Rh FRhK4 cells were pre-treated with equal amounts of VSV-G pseudotyped HIV-2 GH123, SIVmac239, and their derivative viruses. The pretreated cells were then infected with VSV-G pseudotyped GFP expressing HIV-1 carrying SIVmac239 L4/5 (HIV-1-L4/5S-GFP) [47], since we wanted to exclude the effects of endogenous CypA on GFP-expressing virus in FRhK4 cells. The susceptibility of particle-treated cells to virus infection was determined by the percentage of GFP-positive cells.

**Figure 5:**
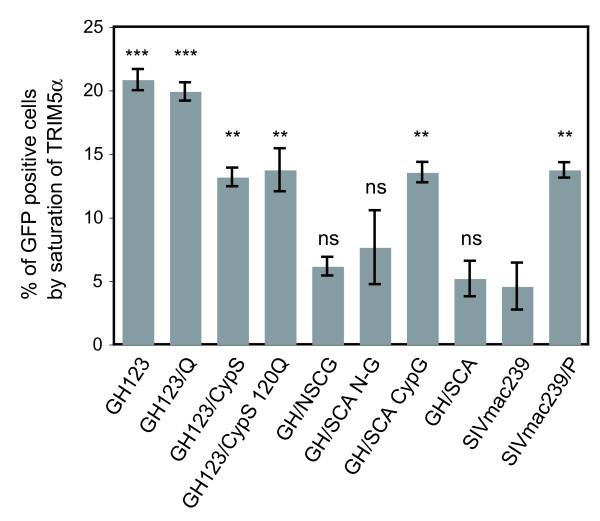
**Activity of GH123, SIVmac239, and their derivatives to saturate TRIM5α in Rh cells**. (A) Rh FRhK-4 cells were pretreated with equal amounts of VSV-G pseudotyped particles (800 ng of p25 or p27) of GH123, GH123/Q, GH123/CypS, GH123/CypS 120Q, GH/NSCG, GH/SCA N-G, GH/SCA CypG, GH/SCA, SIVmac239 or SIVmac239/P for 2 h. Cells were then infected with the VSV-G pseudotyped GFP-expressing HIV-1 vector carrying SIVmac L4/5. Data from triplicate samples (means ± SD) expressed as % GFP positive cells subtracted with the value of mock-treated cells (24.88%) are shown. Statistical significance of differences was calculated using the *t*-test. Asterisks above bars show differences between indicated viruses and SIVmac239. ***, *P *< 0.001; **, *P *< 0.01; ns, not significant. The statistical significance of differences between GH123 and GH123/CypS and that between GH123 and GH/SCA CypG were both < 0.001.

Cells treated with HIV-2 GH123 particles showed enhanced susceptibility to HIV-1 infection compared with non-treated cells (Figure 5), demonstrating that TRIM5α in FRhK4 cells was saturated by the high dose of the particles. In contrast, cells treated with SIVmac239 particles showed very low levels of enhancement. Cells treated with particles carrying GH123/Q showed similar levels of enhanced susceptibility to HIV-1 infection to those of HIV-2 GH123, while cells treated with particles of GH123/CypS, GH123/CypS 120Q, GH/SCA CypG or SIVmac239/P showed intermediate levels of enhancement (Figure 5).

On the other hand, cells treated with particles carrying GH/NSCG, GH/SCA, and GH/SCA N-G showed similar levels of enhancement of HIV-1 susceptibility to those of SIVmac239 (Figure 5). These results are roughly consistent with our data shown in Figures 2 and 3, but there are two differences. First, Rh TRIM5α could completely restrict GH123/CypS and GH123/CypS 120Q (Figure 2), while particles of these viruses showed decreased levels of enhancement compared with those of GH123 or GH123/Q (Figure 5). Second, Rh TRIM5α could slightly restrict GH/SCA N-G (Figure 3E), while particles of this virus failed to saturate Rh TRIM5α (Figure 5). Although the precise reasons for these differences are unclear at present, similar differences were previously reported in HIV-1 CA mutant constructs, and might be due to differences in core stability among mutant viral particles [62]. Nevertheless, our data in Figure 5 clearly indicate the importance of L4/5 (compare GH123 with GH123/CypS, GH/SCA with GH/SCA CypG) and other CA regions (compare GH123 with GH/SCA CypG, SIVmac239 with SIVmac239/P) in the viral ability to saturate TRIM5α in Rh FRhK4 cells, and suggest that the multiple sites in the N-terminal half of GH123 CA affect its binding to Rh TRIM5α.

**Figure 6:**
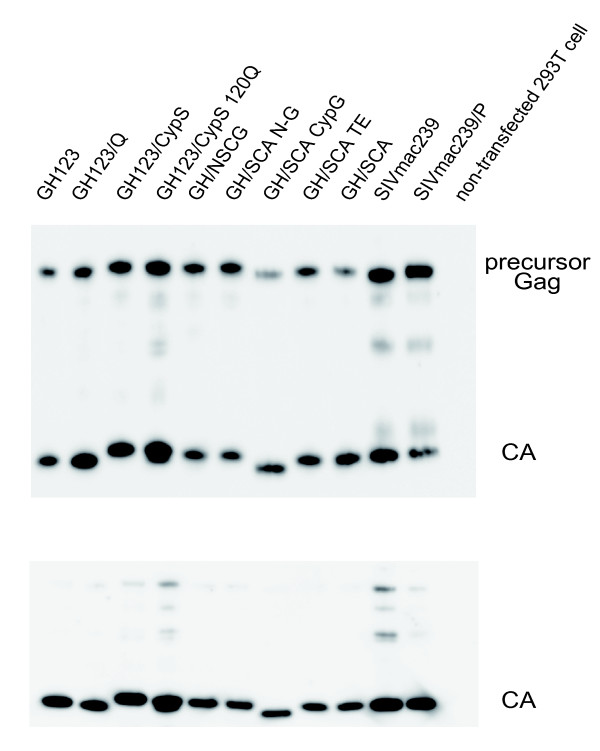
**Western blot analysis of lysates of viral producer cells and viral particles**. Viral proteins in the lysate of equal number of viral producer cells (upper panel) and particle fraction of equal volume of culture supernatant of viral producer cells (lower panel) were visualized by WB using serum from an SIV-infected monkey.

Finally, we checked viral release and maturation/processing of GH123, SIVmac239, and their derivative viruses by a western blot for the lysate of viral producer cells (Figure 6, upper panel) and viral particles (Figure 6, lower panel), since viral maturation is essential for TRIM5α recognition. CA proteins in the cells and released viral particles were clearly detected. CAs with SIVmac239 L4/5 showed slightly reduced mobility compared with those with GH123 L4/5. Although there were small differences in the amounts of CA among viruses tested, there was no difference in the ratio of intracellular CA to those in the released viral particles. It should be also mentioned that there was no difference in the ratio of Gag precursors to processed CA in the viral producer cells. These results indicated that viral release and maturation/processing of the derivative viruses occurred normally.

### Structural model of HIV-2 GH123 CA

**Figure 7:**
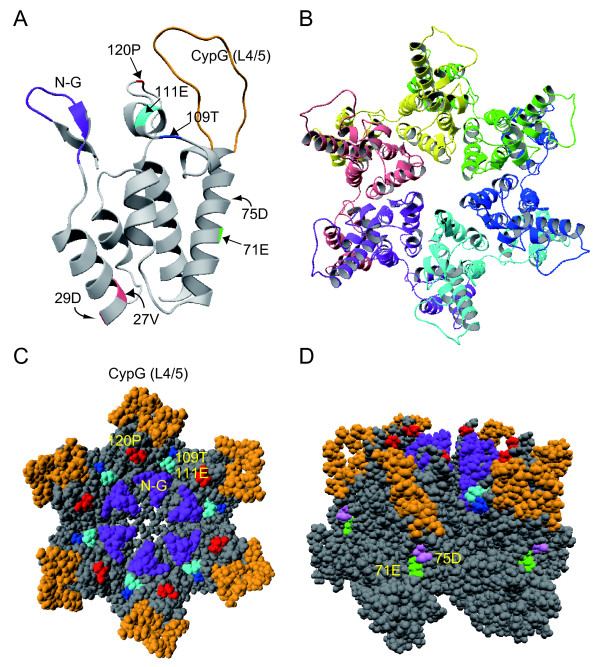
**Three-dimensional structural models of GH123 CA**. (A) Structure of the N-terminal half of CA monomer. The model was constructed by homology-modeling using "MOE-Align" and "MOE-Homology" in the Molecular Operating Environment (MOE) as described previously [73,74]. N-G, dark purple; the 27^th^V and the 29^th^D, pink; Cyp G (L4/5), orange; the 71^st^E, green; the 75^th^D, light purple; the 109^th ^T, dark blue; the 111^th ^E, light blue; and the 120^th ^P, red. The structure of CA hexamer from the top (B and C) and side (D) is shown.

To gain a structural insight into the mechanisms by which Rh TRIM5α recognizes HIV-2 CA, three-dimensional (3-D) models of monomeric and hexameric HIV-2 GH123 CA were constructed using homology-modeling based on the crystal structures of the HIV-2 CA N-terminal domain [48], HIV-1 CA C-terminal domain [49], and the hexameric HIV-1 CA [50]. All amino acid residues conferring sensitivity to Rh TRIM5α restriction (N-G, CypG (L4/5), the 109^th ^T, 111^th ^E, and 120^th ^P) are located on the surface of CA (Figure 7A, C and 7D), suggesting that these positions are involved in interaction with Rh TRIM5α. On the other hand, amino acid residues that impaired viral growth in the absence of TRIM5α (27^th ^V, 29^th ^D, 71^st ^E, and 75^th ^D) are located on the side of CA (Figure 7A and 7D). Although we were unable to determine the effect of these amino acid residues on viral sensitivity to Rh TRIM5α restriction, the structural models suggest that these sites are buried inside multimerized CA. It is therefore unlikely that they are involved in the direct interaction of CA with Rh TRIM5α.

## Discussion

A previous study on the recombination between HIV-2 ROD and SIVmac showed that the CA region corresponding to the CypA binding loop of HIV-1 (L4/5) is the determinant for susceptibility to Rh TRIM5α [42]. A subsequent study on HIV-1 and SIVagmTAN showed that the loop between helices 6 and 7 (L6/7) also contributes to Rh TRIM5α susceptibility [63]. In the present study, we showed that the L4/5 and the 120^th ^amino acids located in L6/7 were required but not sufficient for HIV-2 to evade Rh TRIM5α-mediated restriction.

In addition to L4/5 and L6/7, we found that the N-terminal portion (from the 5^th ^to 12^th ^amino acid residues), and 107^th ^and 109^th ^amino acid residues in α-helix 6 of SIVmac239 CA are required for Rh TRIM5α evasion. The 3-D models of CA showed that the analogous regions of GH123 CA are located on the surface of the CA core structure, suggesting that these sites are involved in the direct interaction of CA with Rh TRIM5α. Our results are in good agreement with a previous report in which the HIV-1 derivative with an entire CA and Vif of SIVmac239 could replicate in Rh cells [64]. In addition, we observed that the HIV-1 derivative with L4/5 and L6/7 of CA and Vif of SIVmac239 (NLScaVR6/7S) that replicates in CM cells [47] failed to replicate in Rh cells (Kuroishi et al., unpublished data).

The growth ability of GH123 was higher than that of SIVmac239 in SeV-infected MT4 cells, but that of many GH123 derivatives with SIVmac239 CA sequences was lower than that of the parental GH123 and comparable with that of SIVmac239 (Figures 1, 2, and 3). However, GH/SCA VD replicated very poorly and GH/SCA ED did not replicate at all. These results were reproducible using the viruses produced with independent plasmid clones, after which Gag processing of these viruses occurred normally (data not shown). As shown in Figure 7, the 27^th ^V and 29^th ^D are in α-helix 1, and the 71^st ^E and 75^th ^D are in α-helix 4. It is possible that the amino acid changes at these sites are harmful for the formation of a multimerized viral core. Supporting this notion, the 27^th ^V and 71^st ^E are highly conserved among different HIV-2 strains in the Los Alamos sequence database. Furthermore, the 71^st ^E and 75^th ^D are located on the lateral side of the CA hexametric structure (Figure 7D), and thus it is possible that these amino acid residues associate with the neighboring CA hexamer. It is thus interesting to know the impact of such amino acid changes on viral core formation.

It has been reported that the CypA-CA interaction renders HIV-1 more susceptible to Rh TRIM5α restriction [65-68]. We found that HIV-2 CA L4/5 corresponding to the CypA binding loop of HIV-1 had the biggest impact on Rh TRIM5α susceptibility, although we could not detect CA-CypA binding (Figure 4). Braaten *et al*. also reported that neither HIV-2 nor SIV recruits CypA into their cores, and that drugs that block CA-CypA interaction have no effect on the titers of these viruses [57]. CA crystal structures of human T-cell lymphotropic virus type 1 [PDB: 1QRJ] [69] and equine infectious anemia virus [PDB: 1EIA] [70] possess an exposed loop directed to the surface of the CA core structure, similar to the HIV-1 CypA binding loop, while retroviruses such as B-tropic murine leukemia virus [PDB: 3BP9] [71] and Jaagsiekte sheep retrovirus [PDB: 2V4X] [72] do not. It is reasonable to assume that this HIV-2 loop would interact with certain host factors other than CypA, and consequently is an attractive target for TRIM5α.

**Figure 8:**
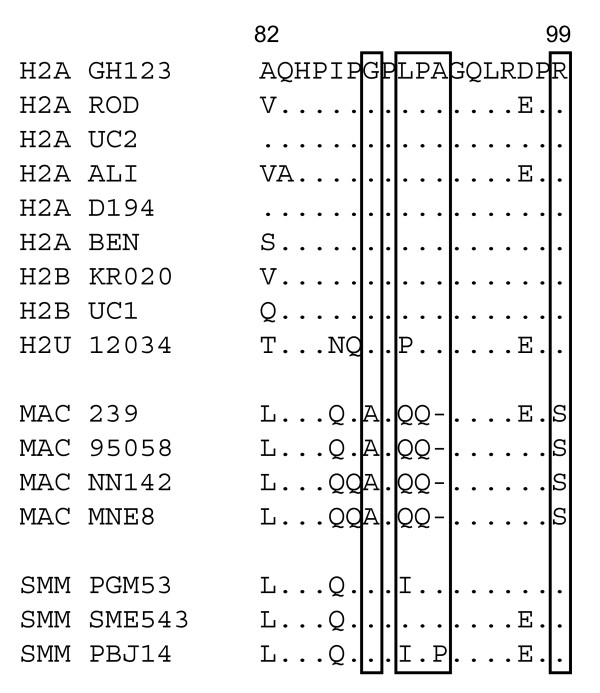
**Alignments of amino acid sequences of the CA L4/5 region of HIV-2, SIVmac, and SIVsmm selected from the Los Alamos databases**. Dots denote the amino acid identical to one of the GH123 CA and dashes denote lack of an amino acid residue that is present in GH123 and other viruses. Boxes show the site of SIVmac-specific amino acid residues. H2A, B, and U represent HIV-2 group A, B, and U, respectively. MAC represents SIVmac, and SMM denotes SIVsmm.

The differences in the L4/5 amino acid sequence among different strains of HIV-2, SIVmac, and SIVsmm are shown in Figure 8. Of these, SIVmac-specific amino acid residues are the 88^th ^A, 90^th^-QQΔ-92^nd^, and 99^th ^S (Figure 8 boxes). Ylinen *et al*. reported that SIVmac QQ LPA, the mutant SIVmac containing HIV-2-specific LPA instead of QQ at the 90^th ^to 92^nd ^positions, was still not restricted by Rh TRIM5α [42], suggesting that the 88^th ^and 99^th ^amino acids or all amino acid substitutions in L4/5 between SIVmac and HIV-2 are involved in resistance to Rh TRIM5α restriction.

We previously reported that the TFP motif in the SPRY domain of Rh TRIM5α is important in restriction of HIV-2 strains that are not restricted by CM TRIM5α [34]. In the present study, we confirmed that this motif is both necessary and sufficient to restrict various HIV-2-SIVmac chimeras that are restricted by Rh TRIM5α. If the TFP motif in the SPRY domain of Rh TRIM5α is directly involved in interaction with viral CA, it is not clear why multiple regions of SIVmac239 are necessary for evasion from TRIM5α with a TFP motif. We previously constructed the 3-D structural model of the SPRY domain [36] using homology modeling. It would therefore be of interest to construct a 3-D binding model of CA and TRIM5α, and to understand how the 339^th^-TFP-341^st ^motif of Rh TRIM5α affects recognition of the CAs that differ at multiple positions.

## Conclusion

We found that multiple regions of the SIVmac CA, not only L4/5 and the 118^th ^amino acid but also the N-terminal portion (from the 5^th ^to 12^th ^amino acid residues), and the 107^th ^and 109^th ^amino acid residues, are necessary for complete evasion from Rh TRIM5α restriction.

## Competing interests

The authors declare that they have no competing interests.

## Authors' contributions

KK and HS performed experiments. EEN and TS participated in its design. MY and HS carried out computational analysis. KK, EEN, HS and TS drafted the manuscript. All authors read and approved the final manuscript.

## Authors' information

KK is a research fellow of the Japan Society for the Promotion of Science. HS was a PhD student of Osaka University. HS is a chief of Laboratory of Viral Genomics, Pathogen Genomics Center, National Institute of Infectious Diseases, Japan; and MY is a staff of this laboratory. TS is a professor, and EEN is an assistant professor of Research Institute for Microbial Diseases, Osaka University.
